# Dr. William Buckingham Canfield

**Published:** 1900-02

**Authors:** 


### OBITUARY.

Dr. William Buckingham Canfield, of Baltimore, Md., died at Roosevelt Hospital, New York, December 27, 1899, aged 42 years. The circumstances resulting in his death are extremely sad. He went to New York about a month before his decease apparently in good health. He fell to the pavement November 27th and became immediately unconscious. After removal to the hospital he improved somewhat, enough to recognise relatives who had been summoned, but relapsed and died on the day mentioned, of fracture of the skull.

Dr. Canfield was one of the most promising of the younger members of the Baltimore profession. He became known outside his city as an author and at the time of his death was editor of the *Maryland Medical Journal*. The news of this sad event was a great shock to his many friends, all of whom cherish for his memory the highest regard.

